# Azobenzene Photoswitching with Near-Infrared Light Mediated by Molecular Oxygen

**DOI:** 10.1021/acs.jpcb.1c08012

**Published:** 2021-11-04

**Authors:** Kim Kuntze, Jussi Isokuortti, Antti Siiskonen, Nikita Durandin, Timo Laaksonen, Arri Priimagi

**Affiliations:** Faculty of Engineering and Natural Sciences, Tampere University, P.O. Box 541, FIN-33101 Tampere, Finland

## Abstract

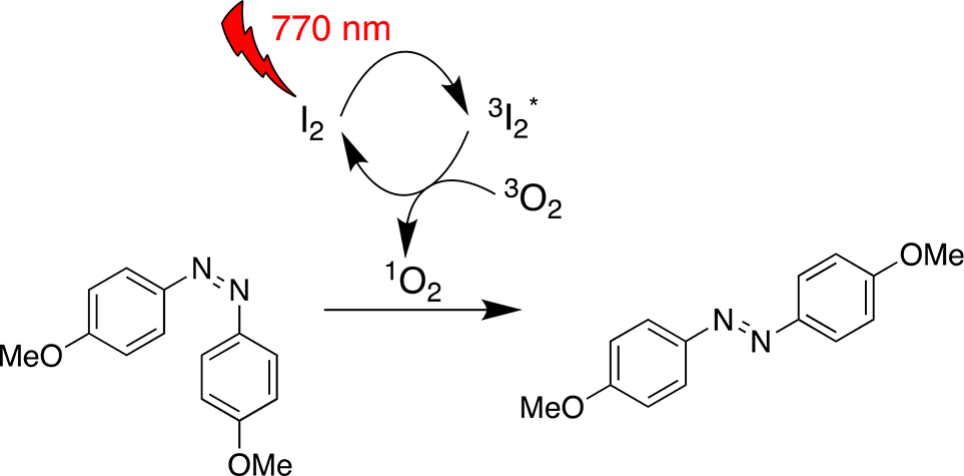

Efficient photoisomerization between the cis and the trans states of azobenzenes using low-energy light is desirable for a range of applications in, e.g., photobiology yet challenging to accomplish directly with modified azobenzenes. Herein, we utilize molecular iodine as a photocatalyst to induce indirect cis-to-trans isomerization of 4,4′-dimethoxyazobenzene with 770 nm near-infrared light, showing robustness during more than 1000 cycles in ambient conditions. Intriguingly, the catalysis is mediated by molecular oxygen, and we demonstrate that other singlet-oxygen-generating photosensitizers besides iodine, i.e., palladium phthalocyanine, catalyze the isomerization as well. Thus, we envision that the approach can be further improved by employing other catalysts with suitable photoelectrochemical properties. Further studies are needed to explore the applicability of the approach with other azobenzene derivatives.

## Introduction

Molecular iodine is known to catalyze organic reactions when illuminated with visible light. The list of iodine-photocatalyzed reactions includes the oxidation of tertiary amines,^1^ allylic and benzylic alcohols,^2,3^ and styrenes^4^ into the respective aldehydes and has recently been extended with the degradation of trichlorophenol^5^ and selected intra- or intermolecular metal-free coupling reactions.^6−9^ In most reactions, the catalytic activity is attributed to iodine radicals formed in the homolysis of the I–I bond, presumably after initial excitation to ^3^I_2_*.^1−5,8^ Also ionic pathways have been reported in which the triplet excited state of iodine functions as a singlet oxygen sensitizer^10^ and the singlet oxygen in turn regenerates the iodine at the end of the catalytic cycle.^6−9^ The photochemistry of iodine has been studied over many decades,^11−19^ and it is known that the absorption of molecular iodine in the visible range is attributed to overlapping electronic transitions to three excited states: bound triplet states A and B and an unbound singlet state C (Figure S1).^17^ The C ← X and B ← X transitions account for the absorption in most of the visible range, and although the B state is bound, it is crossed by unbound states. This explains the observed dissociation of the iodine molecule when excited with blue or white light.^11,13,20^ However, the A state, reached by irradiation with longer wavelengths (>650 nm), is not crossed by unbound states. Recombination of iodine atoms may result in a slightly lower energy A′ state but does not lead to unbound states either.^17^ Therefore, excitation at long wavelengths can lead to a fairly stable triplet-excited (A or A′ state) iodine molecule which may act as a donor in triplet energy transfer (TET). Even though excitation inside the therapeutic optical window (>650 nm) would be greatly beneficial for biological applications, no studies on iodine catalysis using red or near-infrared light excitation have been performed.

An important photochemical reaction that iodine is known to photocatalyze is the cis–trans isomerization of azobenzenes,^21,22^ a photoswitch family utilized in photoresponsive pharmaceuticals,^23,24^ catalysts,^25^ and materials.^26−29^ These applications benefit from precisely controlled light-driven isomerization, preferably with low-energy (red/near-infrared) light.^30^ Unfortunately, the lifetime of the metastable cis isomer is typically short for red-light-absorbing azobenzenes, an undesired feature in most applications. This deficiency can be addressed by synthetic modifications, especially ortho substitution with certain moieties^31−35^ that stabilize the cis isomer and in some cases separate the low-energy *n*–π* absorption bands of the isomers, allowing selective trans–cis and cis–trans photoisomerization with visible light. These bands are, however, limited to wavelengths below 600 nm with low molar absorptivity in the red end of the visible spectrum. Suitable photocatalysts offer an alternative pathway to control the photoswitching with low-energy irradiation. Azobenzenes have a short-living triplet state whose excitation leads mainly to trans-azobenzenes via rapid intersystem crossing.^22,36−42^ Thus, with a suitable triplet sensitizer it is possible to accelerate the cis–trans isomerization using significantly longer wavelengths than those absorbed by the azobenzene.^39,43^ However, isomerization via TET suffers from sensitivity toward oxygen, hampering the functionality of the systems in ambient conditions. If the redox potentials of the *cis*-azobenzene and the sensitizer are matched, photoinduced electron transfer (PET) becomes feasible, and both reductive^44^ and oxidative^45^ PET processes have been used to drive the isomerization with wavelengths as long as 660 nm in the latter case. Despite recent progress in the field, iodine-catalyzed photoisomerization has not been studied since the first reports in the 1960s and 1970s. Therein, either 545 nm green light excitation of iodine or ultraviolet light excitation of a charge transfer complex between iodine and azobenzene was utilized, neglecting the possibility of low-energy-light excitation.^21,22^ Yet iodine would be highly attractive for this purpose because of its low cost, nontoxic nature, environmental friendliness, and, above all, the potential of catalyzing the isomerization in response to NIR light in ambient conditions. In this study, we set out to explore the photocatalytic properties of molecular iodine with the aim of controlling the azobenzene isomerization with low-energy light while also broadening the utility of molecular iodine in other photocatalytic processes.

## Results and Discussion

**Figure 1:**
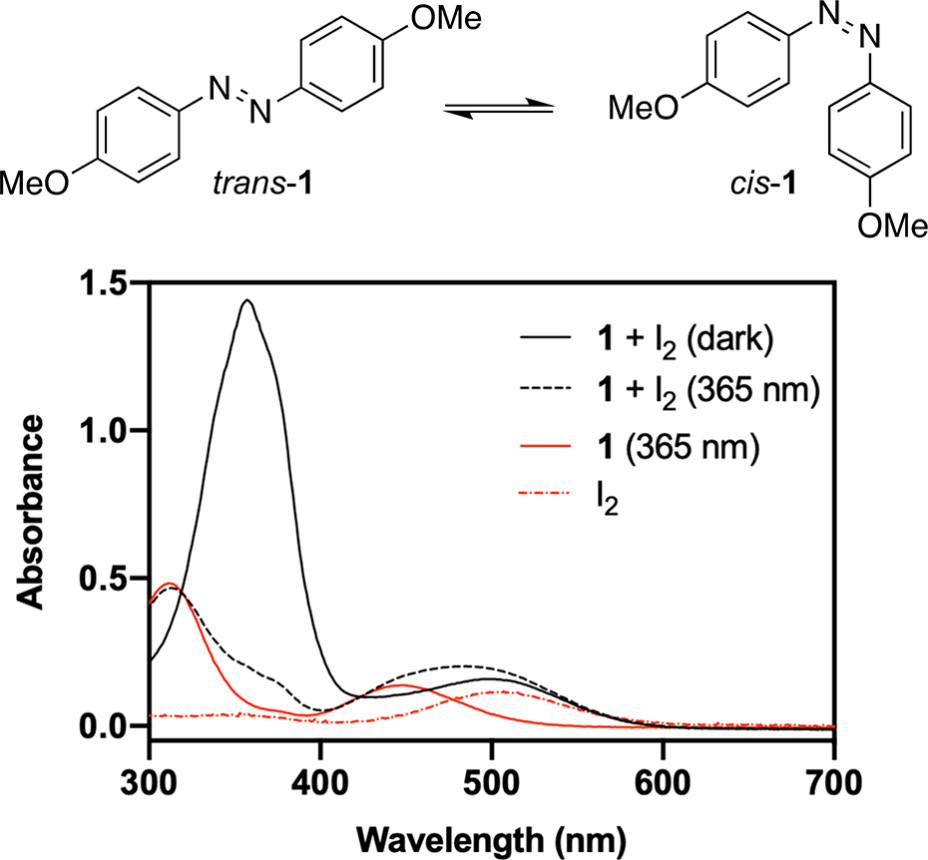
Absorption spectra of **1** (50 μM) + I_2_ (200 μM) in the dark and upon illumination with UV light as well as pure **1** and iodine in dichloromethane.

Initially, we restricted our studies to the moderately electron-rich 4,4′-dimethoxyazobenzene (**1**) that has a relatively stable cis isomer (half-life 13 h) and has been found to isomerize efficiently with triplet sensitizers and photoinduced electron transfer agents without considerable fatigue.^45^ Solvents with nucleophilic electron pairs blue shift the absorption spectrum of iodine, so we studied the isomerization in three inert solvents of varying density and polarity: dichloromethane (DCM), carbon tetrachloride (TCM), and *n*-hexane (hexane). First, we recorded and analyzed the absorption spectra of **1** and iodine separately and in one solution (Figure 1). The absorption spectrum of *trans*-**1** is governed by an intense band at ca. 370 nm, corresponding to the π–π* transition. Upon irradiation with 365 nm light, a photostationary state of >95% *cis*-**1** is acquired. Both *trans*-**1** and *cis*-**1** also exhibit a less intense *n*–π* band between 400 and 600 nm, but neither isomer absorbs at >650 nm. The absorption band of iodine, on the other hand, reaches well beyond the visible range: absorption can be observed even at >800 nm with a more concentrated sample (Figure S2). The absorption spectrum of the *cis*-**1** + I_2_ mixture is the sum of the spectra of *cis*-**1** and I_2_ with no indication of additional absorption bands corresponding to a charge transfer complex.

**Figure 2:**
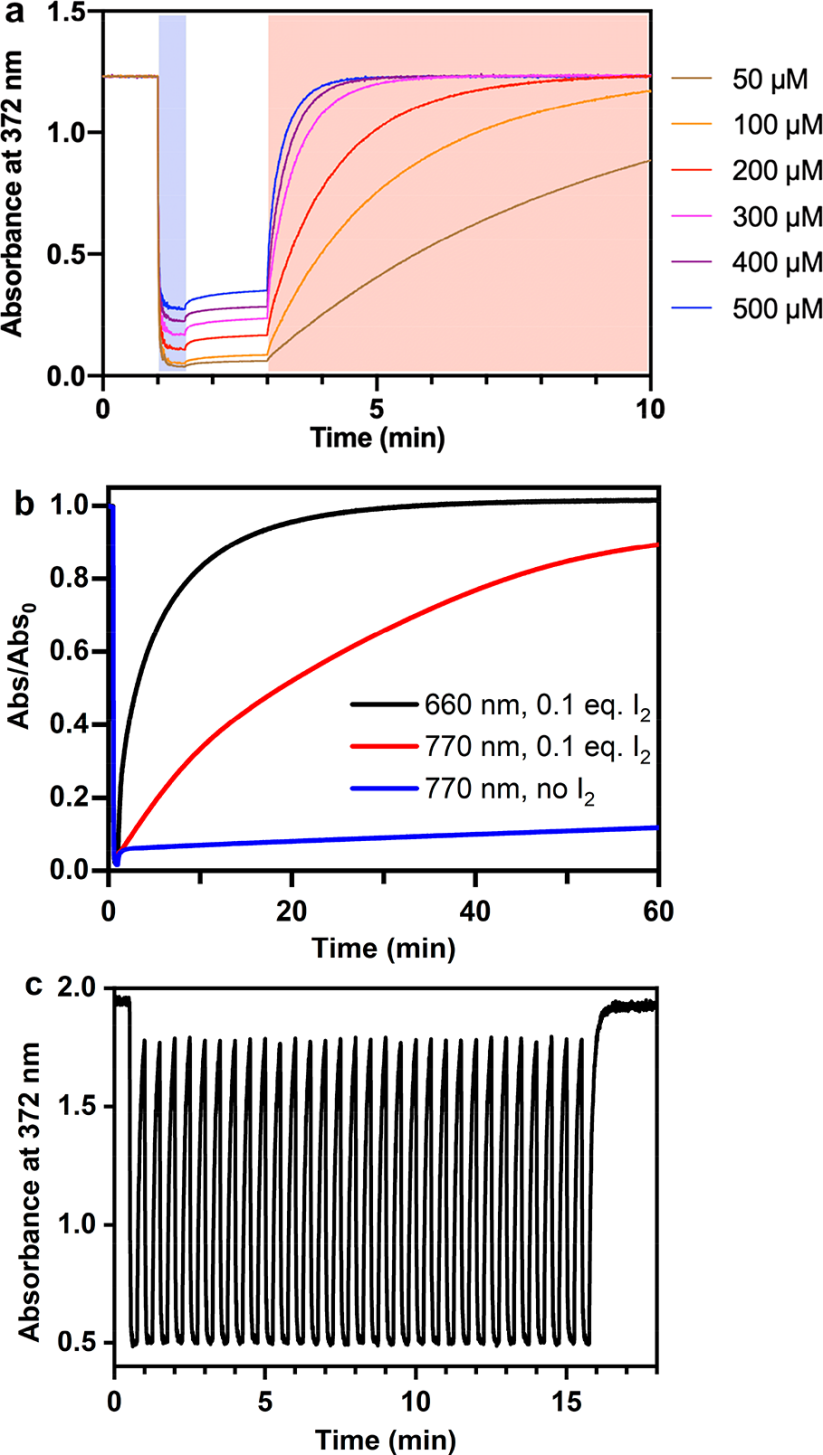
(a) Photoisomerization curves of **1** in DCM with 0–10 equiv of iodine. Illumination with 365 and 770 nm is shown in light purple and red shading, respectively. (b) Photoisomerization curves of **1** in DCM with 0.1 equiv of iodine under illumination with 660 and 770 nm light. (c) Cycles of 365 and 770 nm illumination (∼15 s).

Having established that in the red/NIR region the only absorbing species is molecular iodine and the excitation should result in ^3^I_2_* in a nondissociative manner, we proceeded to study the photoisomerization with varying iodine concentrations. This was monitored by recording the absorbance value near the maximum of the π–π* band of *trans*-**1** (372 nm). Starting from *trans*-**1**, a *cis*-**1**-rich mixture was reached by illuminating the mixture with 365 nm light (Figure 2a), which is seen as a drop in the absorbance. The cis fraction in the photostationary state decreased with increasing iodine concentration due to competing absorption by the iodine molecule and subsequent iodine-catalyzed cis-to-trans isomerization. When the irradiation was stopped, the absorbance increased slightly, mostly due to diffusion from the unilluminated parts of the solution as thermal isomerization is too slow to be observed in this time scale. Upon illumination with 770 nm near-infrared light, cis-to-trans isomerization took place rapidly. Initially, we used 1–10 equiv of iodine to ensure fast photoisomerization, as the molar absorptivity of iodine is very low in the NIR region (ε_770_ = 8.6 M^–1^ cm^–1^). However, the catalysis worked perfectly with only 0.1 equiv of iodine (Figure 2b, Figure S3). In addition to using higher iodine loadings, the rate can be accelerated by a factor of 4.8 using red instead of NIR light (ε_660_ = 41 M^–1^ cm^1^), still keeping the excitation wavelength well within the therapeutic optical window. An increase in the rate by approximately this factor (4.4) was indeed observed when switching from 770 to 660 nm excitation (Figure 2b, Table S1), although comparison between different excitation wavelengths is hampered by the differences in the intensities and spectral shapes of the light sources. Furthermore, we would like to highlight the robustness of the system: virtually no photobleaching was observed over the course of 30 switching cycles under ambient conditions (Figure 2c), and the system was fully functional even after 1000 cycles (Figure S4).

**Figure 3:**
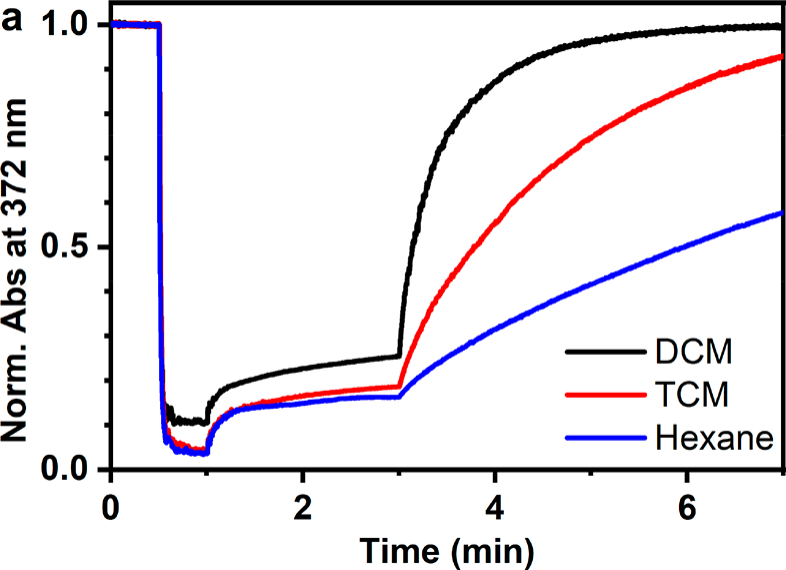
Photoisomerization curves of **1** with 4 equiv of iodine in dichloromethane, carbon tetrachloride, and *n*-hexane. Illumination with 365 nm at 0.5–1.0 min and with 770 nm from 3.0 min onward.

Three mechanistic pathways can give rise to the iodine-catalyzed photoswitching: (i) formation of I^•^ radicals and subsequent radical mechanism,^21^ (ii) triplet energy transfer, and (iii) photoinduced electron transfer. To distinguish between these, we first studied the effect of the solvent. The photocatalyzed reaction proceeded fastest in dichloromethane and slowest in hexane (Figure 3, Table S1). The fact that the catalysis is faster in carbon tetrachloride than that in hexane (by a factor of 3.2) speaks against the I^•^ mechanism, as the photodissociation rate should be ca. 5 times higher in hexane than that in TCM.^20^ In addition, the dissociation reaction is unlikely in the NIR region.^12^ TET is ruled out by the observation that the rate is 2.2-fold in dichloromethane compared to carbon tetrachloride in which ε_770_ is higher (Figure S2) and the triplet state lifetime of iodine is longer, and, thus, TET should be more efficient.^17^ This hints toward the formation of a charged species during the process. PET seems probable as electron transfer processes are polarity dependent.^46^ We note that the observed solvent effect is not attributed to the solubility of oxygen, which is highest for *n*-hexane and lowest for dichloromethane.^47,48^

**Figure 4:**
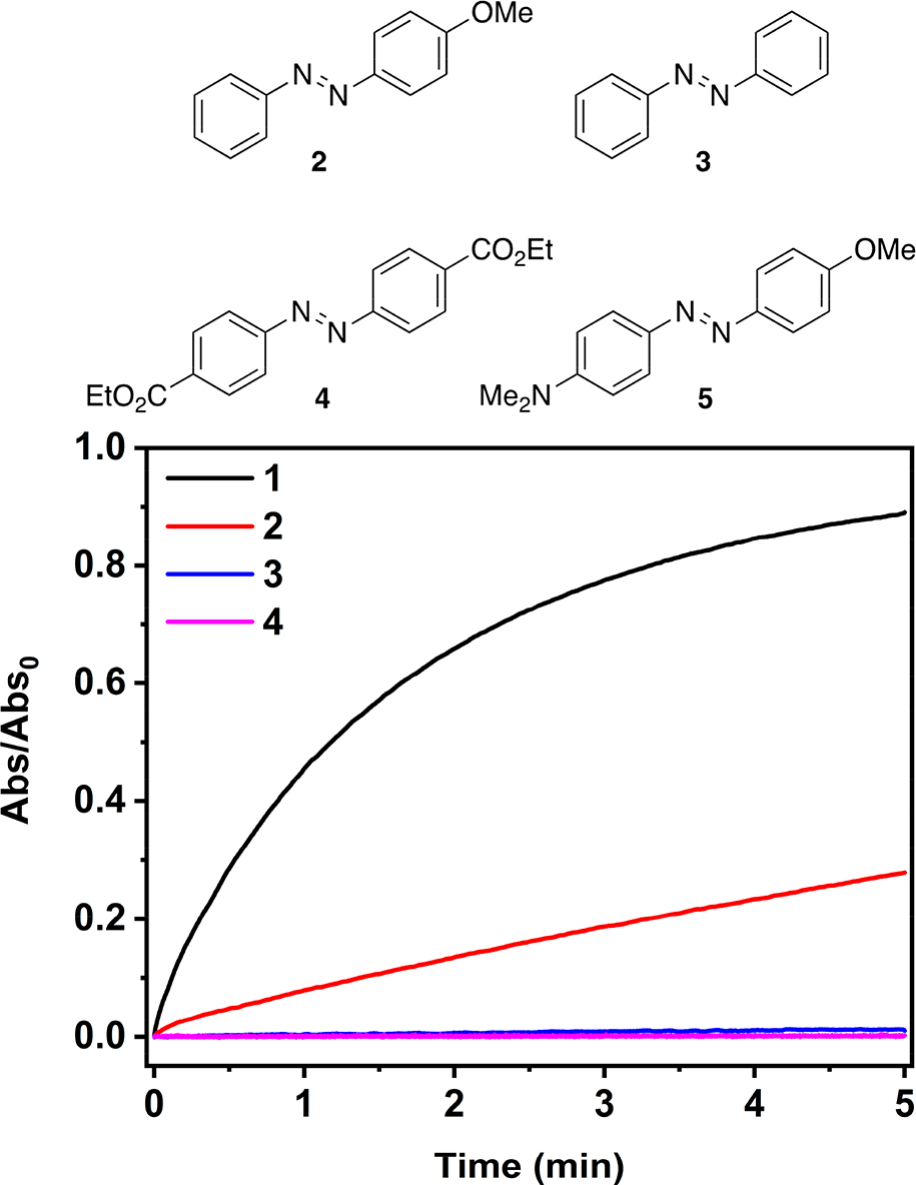
Photoisomerization curves of **1**–**4** with 4 equiv of iodine in dichloromethane. Illumination with 770 nm starting at 0 min; trans-to-cis isomerization with 365 nm is omitted for clarity. Vertical axis shows the absorbance relative to the initial value, i.e., pure trans isomer.

Both oxidative and reductive PET processes are feasible, but for an electron-rich azobenzene such as **1**, the oxidative pathway is more probable.^45^ We also screened the reaction for three other less electron rich azobenzenes: 4-methoxyazobenzene **2**, unsubstituted azobenzene **3**, and an azobenzene diester **4**. The photocatalysis did not take place for **3** and **4** and was pronouncedly slower for **2** (Figure 4). This is another indication of an oxidative process. We also tried to test the reaction on a more electron-rich azobenzene **5** but were unable to induce the trans-to-cis photoisomerization upon excitation with 405 nm light, probably due to a ground-state interaction between **5** and iodine that blue shifts the absorption spectrum of iodine (Figure S5).

**Figure 5:**
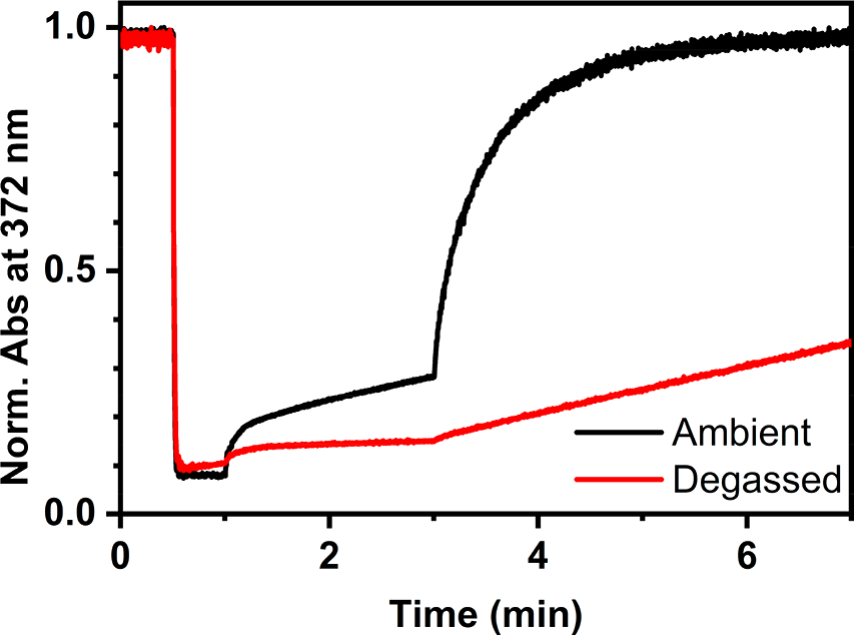
Photoisomerization curves of **1** with 4 equiv of iodine in dichloromethane at ambient conditions and after freeze–pump–thaw deaeration. Illumination with 365 nm at 0.5–1.0 min and with 770 nm from 3.0 min onward.

We also carried out the same experiment in nitrogen/argon-purged and freeze–pump–thaw-deaerated solutions, as both PET and TET systems are typically sensitive to oxygen.^43,45^ To our great surprise, photoisomerization was pronouncedly *slower* in the partly (see SI) deoxygenated solutions than that under ambient conditions (Figure 3, Table S1), contrary to earlier TET and PET studies.^43,45^ This indicates a central role for oxygen in the mechanism. As triplet iodine is capable of sensitizing singlet oxygen,^10^ which in turn has been shown to isomerize alkenes,^49^ we investigated the possibility of a singlet-oxygen-mediated route (Figure 5). Our attempts to use oxygen scavengers failed due to ground-state interaction with iodine (Figure S6), and similar results are expected with any scavenger containing nucleophilic electron pairs. Hence, we probed the possibility of a singlet-oxygen-mediated route with palladium octabutoxyphthalocyanine (PdPc), an efficient singlet oxygen sensitizer with a low triplet energy (1.13 eV) and short lifetime (3.5 μs) and thus low probability of direct TET to **1** to occur.^50^ We first chose the concentration of PdPc (0.36 μM) so that its optical density at 770 nm was matched with that of a 200 μM iodine solution. Thus, [^1^O_2_] should be higher for the PdPc solution as it has a higher quantum yield of singlet oxygen generation.^10,50^ Almost no photocatalyzed isomerization was observed (Figure S7). With an equimolar (50 μM) concentration of PdPc, however, the cis-to-trans isomerization was relatively rapid, albeit slower than that for an equivalent amount of iodine (Figure S8). Most importantly, PdPc was also found to function only in the presence of oxygen (Figure S7), ruling out any direct TET or PET processes. These findings reveal that (i) singlet oxygen indeed plays a key role in the mechanism, as this is the only known product of the reaction between excited PdPc and oxygen, and (ii) iodine must have another role besides generating singlet oxygen, since the iodine-catalyzed reaction is faster than the equivalent PdPc-catalyzed reaction even though PdPc undisputedly produces more singlet oxygen to the solution. Thus, the PET most probably takes place between singlet oxygen (a strong oxidant^51^) and **1**.

*Combining these results, we propose an oxygen-mediated PET mechanism for the catalysis, as illustrated in eqs 1–7. After the initial excitation of I_2_ to ^3^I_2_* (eq 1), the triplet excited species sensitizes the formation of singlet oxygen ^1^O_2_ (eq 2), which then oxidizes **1** (eq 3).^9^ The formed radical cation *cis*-**1**^•+^ has an extremely low isomerization energy barrier, rapidly yielding *trans*-**1**^•+^ (eq 4).^45^ Electron transfer from O_2_^•–^ to *trans*-**1**^•+^ then terminates the cycle (eq 5). All of these reactions are also possible for PdPc.
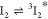
1
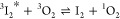
2
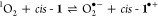
3
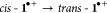
4
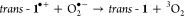
5In the case of iodine, additional steps are possible. The termination step can proceed via electron transfer from O_2_^•–^ to iodine (eq 6)^52^ and then to *trans*-**1**^•+^ (eq 7). In addition, it is possible that the oxygen formed when interacting with iodine is singlet excited, further accelerating the reaction.^53^ These additional routes could explain the markedly higher rate when using iodine as compared to PdPc. We also considered the possibility of iodine dissociation upon interaction with the sensitized singlet oxygen^54^ and subsequent I^•^ radical mechanism but ruled it out as it did not explain the solvent polarity dependence. However, we want to highlight that more mechanistic studies, both experimental and computational, should be carried out for further verification of this plausible mechanism.
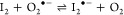
6
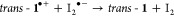
7

## Conclusions

We have shown that singlet oxygen generated upon NIR light excitation of molecular iodine or another sensitizer can be used to induce robust azobenzene photoswitching. In fact, and most interestingly, the catalytic cycle is dependent on molecular oxygen, a feature unknown for any photocatalytic systems operating on photoswitches. Noteworthy, practically no photobleaching was observed after 1000 cycles. This is in stark contrast to our recently published TET-based NIR light catalysis, where molecular oxygen was detrimental for the performance of the azobenzene catalytic system. We envision that our concept can be further developed with other perhaps even more efficient NIR-absorbing singlet oxygen sensitizers with the same or better photoelectrochemical properties than iodine and PdPc. Moreover, our approach provides inspiration for future iodine photocatalysis also outside the field of photoswitch isomerization. Further studies should be carried out to determine what other azobenzene derivatives (and perhaps other photoswitches) the approach can be applied to. Additional experimental and computational studies are also needed to verify the mechanistic pathway of the catalysis.

## Supporting Information Available

Experimental section, synthesis and characterization of the studied materials, deaeration procedures, fitted reaction rates, other supporting results (PDF)
