# Supplementary material for: Azobenzene Photoswitching with Near-Infrared Light Mediated by Molecular Oxygen
Source: J Phys Chem B. 2021 Nov 4;125(45):12568–73. doi: 10.1021/acs.jpcb.1c08012 (PMC8607416; doi:10.1021/acs.jpcb.1c08012)
Supplement: Supplementary file 1 — jp1c08012_si_001.pdf [file jp1c08012_si_001.pdf]

Supporting Information

Azobenzene Photoswitching with Near-Infrared Light Mediated by Molecular Oxygen

Kim Kuntze, Jussi Isokuortti, Antti Siiskonen, Nikita Durandin, Timo Laaksonen and Arri Priimagi*

Faculty of Engineering and Natural Sciences, Tampere University, P.O. Box 541, FIN-33101 Tampere, Finland. E-mail: arri.priimagi@tuni.fi

Efficient photoisomerization between the cis and trans states of azobenzenes using low-energy light is desirable for a range of applications in, e.g., photobiology, yet challenging to accomplish directly with modified azobenzenes. Herein, we utilize molecular iodine as a photocatalyst to induce indirect cis-to-trans isomerization of 4,4'-dimethoxyazobenzene with 770 nm near-infrared light, showing robustness during at least 1,000 cycles in ambient conditions. Intriguingly, the catalysis is mediated by molecular oxygen, and we demonstrate that other singlet-oxygen-generating photosensitizers besides iodine, i.e., palladium phthalocyanine, catalyze the isomerization as well. Thus, we envision that the approach can be further improved by employing other catalysts with suitable photoelectrochemical properties. Further studies are needed to explore the applicability of the approach with other azobenzene derivatives.

Table of Contents

Photochemical measurements	S2
Deaeration of solvents	S2
Supporting results	S3–S10
Materials	S10–S12
References	S13

Photochemical measurements

UV-Visible absorption spectra were recorded with an Agilent Cary 60 spectrophotometer equipped with an Ocean Optics Qpod 2e Peltier-thermostated cell holder whose temperature accuracy is 0.1 °C. Photoexcitation was conducted using a Prior Lumen 1600 light source containing multiple narrow-band LEDs at different wavelengths. The illumination powers (36 mW for 770 nm and 60 mW for 660 nm) were measured with a Coherent LabMax thermal power meter. Quartz fluorescence cuvettes with an optical path of 1.0 cm were used for all measurements except the freeze-pump-thaw-degassed solutions that were measured in custom-made Schlenk cuvettes. The solutions were stirred with a small stirring bar to ensure mixing during irradiation, but despite this, the solutions do not become entirely homogenous in 30 seconds.

Deaeration of solvents

A solution containing 50 μM of **1** and 200 μM of iodine was created and first studied at ambient conditions, after which the same solution was deaerated via five freeze-pump-thaw cycles inside a custom-made Schlenk cuvette. After deaeration the sample was kept under an argon atmosphere during the measurements.

We note that due to the great volatility of DCM, degassing lead to evaporation of the solvent and thus an increase in the concentrations of the solution. In addition, the deaerated solutions should be viewed as partly (not totally) oxygen-free, although the oxygen concentration is relatively low. To estimate the amount of oxygen, we prepared a solution of palladium tetraphenylporphyrin (PdTPP) that has a triplet lifetime of 723 μs in a completely degassed solution, and freeze-pump-thaw deaerated it. From the observed triplet state lifetime (400 μs) the oxygen concentration can be calculated[4,5] using the equation

$$\frac{\tau_0}{\tau} = 1 + k_{diff}\tau_0[O_2]$$

in which τ is the observed lifetime, τ_0 is the lifetime in a completely degassed solution, and k_{diff} is the diffusion rate estimated to be 1×10^{-10} M^{-1} s^{-1}. These values yield an oxygen concentration of 0.11 μM. If the diffusion is estimated to be slower, 5×10^{-9} M^{-1} s^{-1}, an oxygen concentration of 0.22 μM is acquired. Both estimated concentrations are extremely small compared to the ambient value[6] of 11 mM. However, we want to underline that these calculations should be viewed as qualitative: the experiment verifies that the oxygen content is very small (yet it is present) but the exact concentration is not known.

Supporting results

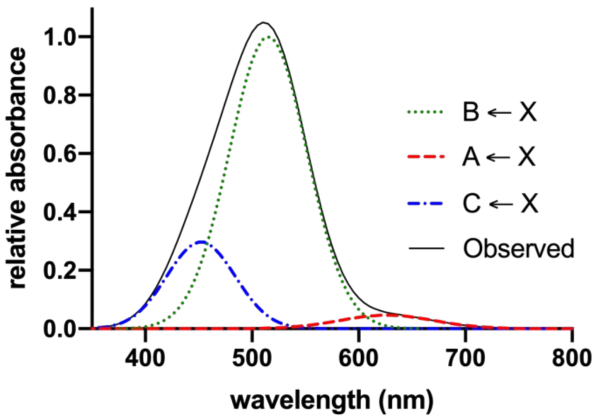

Figure S1. *A schematic presentation of the iodine molecule absorption bands in the visible range, showing that at >650 nm the dominating transition is A ← X.*

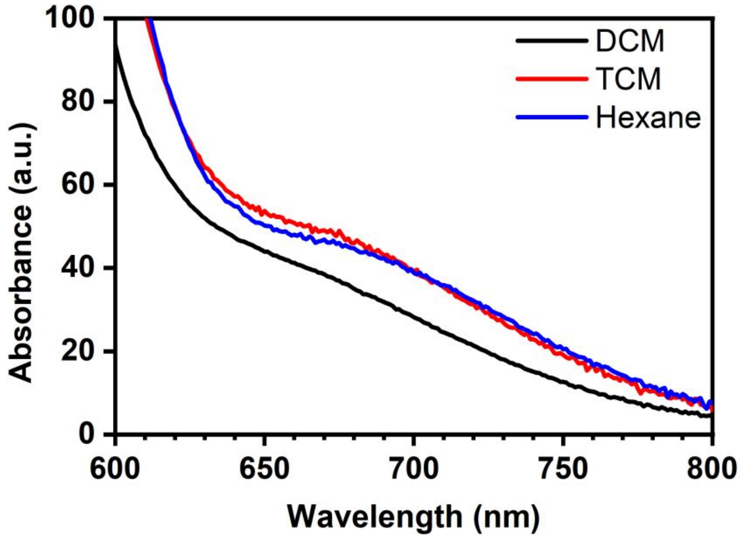

Figure S2. *Absorbance of 5 mM iodine in DCM, TCM and hexane at 600–800 nm, showing that the spectral shape is almost identical in the three solvents, with molar absorptivity of 8.6 dm^3 mol^{-1} cm^{-1} in DCM and 14 dm^3 mol^{-1} cm^{-1} in TCM and hexane at 770 nm.*

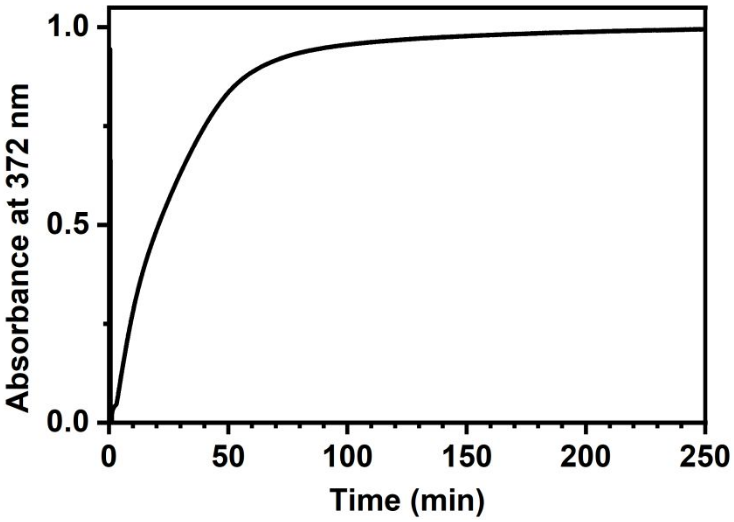

Figure S3. *Photoisomerization curve of **1** (50 µM in DCM) with 5 µM (0.1 eq.) iodine. Irradiation with 365 nm at 0.5–1.0 min and with 770 nm from 3.0 min onward.*

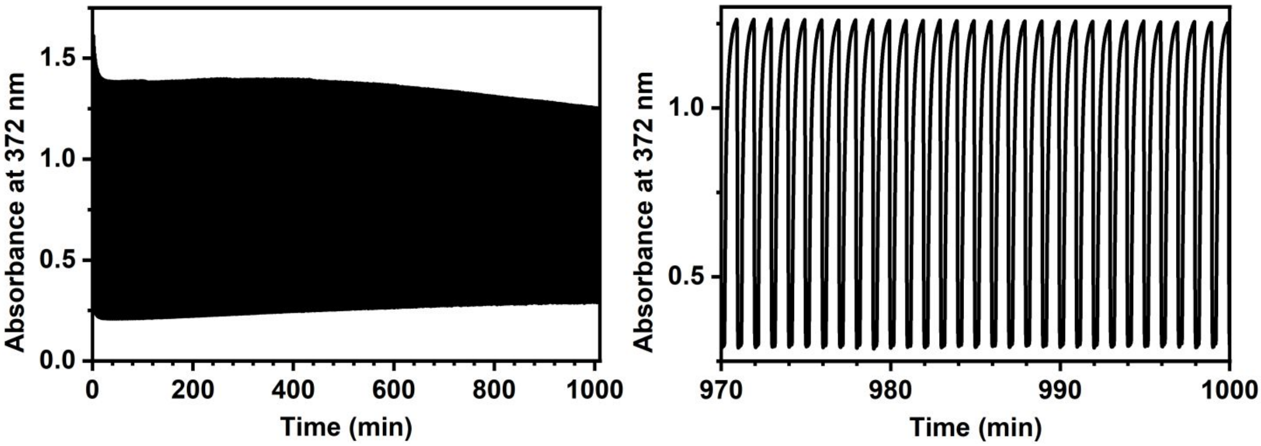

Figure S4. *1,000 cycles of 365 and 770 nm illumination (à 15 and 45 s) and a close-up on the last 30 cycles, showing only slight degradation over time (0.02% average absorbance drop per cycle). We note that upon such a long irradiation period the power of the LED may change, and this may also cause the observed changes in the cycle minima and maxima.*

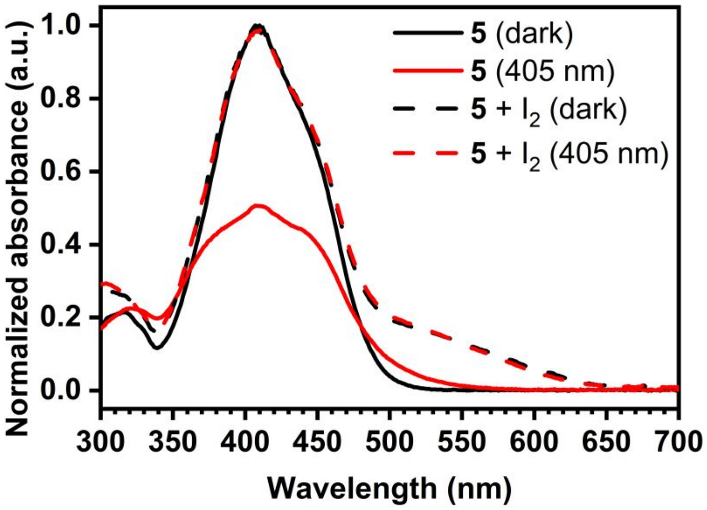

Figure S5. *Absorption spectrum of **5** (50 µM) in dark and upon irradiation with 405 nm light, and **5** (50 µM) and I_2 (200 µM) in dark and upon irradiation with 405 nm light in DCM, showing that the trans-to-cis photoisomerization is disabled when iodine is present.*

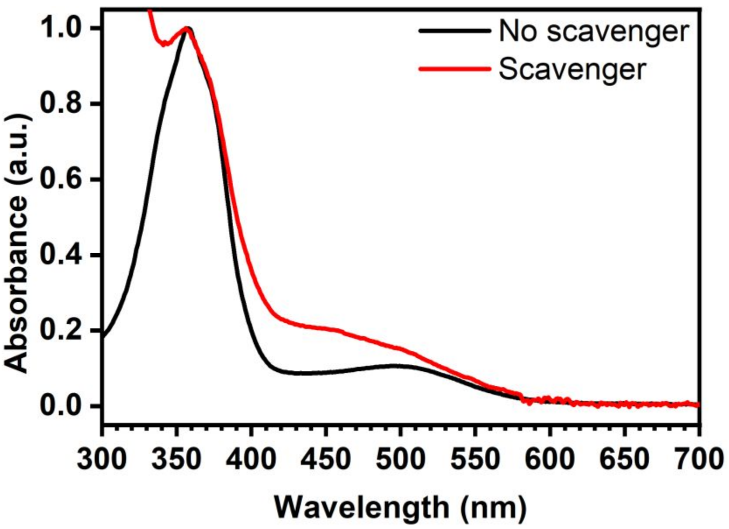

Figure S6. *Absorption spectrum of **1** (50 µM) and iodine (200 µM) in DCM with and without 2,5-dimethylfuran as a singlet oxygen scavenger. Ground state interaction between iodine and furan is observed as an increase in the absorbance at around 450 nm.*

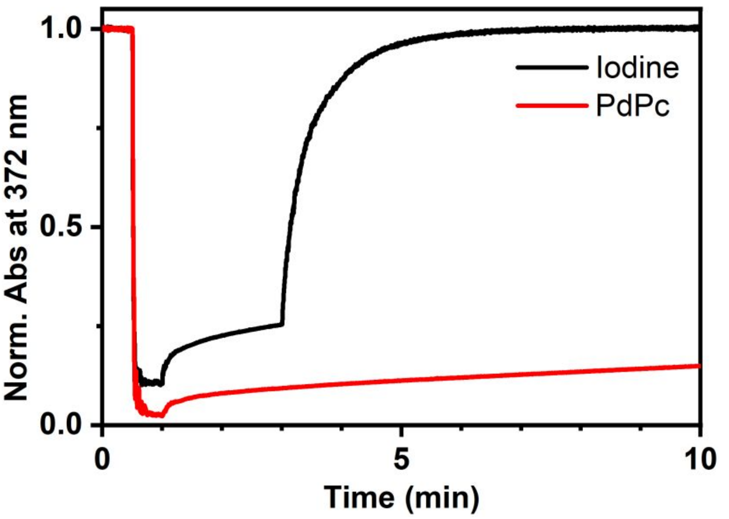

Figure S7. *Photoisomerization curve of **1** (50 µM in DCM) with 200 µM (4.0 eq.) iodine and 0.36 µM (0.72%) PdPc. Irradiation with 365 nm at 0.5–1.0 min and with 770 nm from 3.0 min onward. With these concentrations the optical densities of iodine and PdPc are equal at 770 nm.*

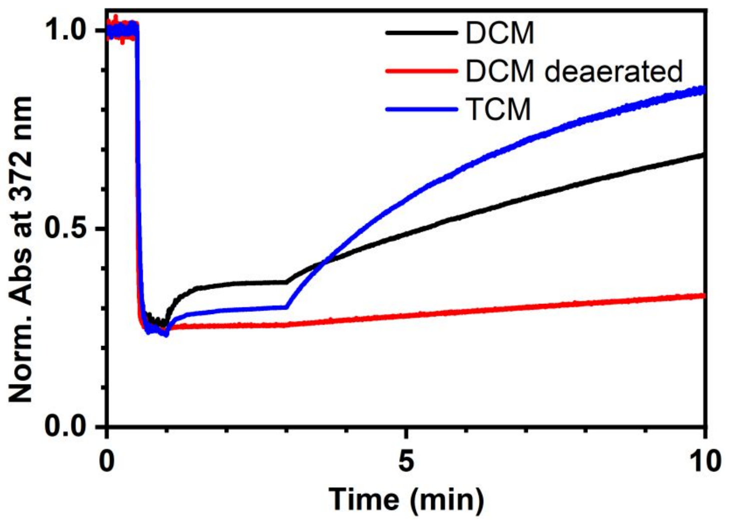

Figure S8. *Photoisomerization curve of* ***1*** *(50 µM) with 50 µM (1.0 eq.) PdPc in ambient DCM, freeze-pump-thaw-deaerated DCM and ambient TCM. Irradiation with 365 nm at 0.5–1.0 min and with 770 nm from 3.0 min onward.*

Isomerization rates were determined by fitting an exponential function ($y = A*exp(x/t)+y_0$) to the isomerization curves (fitting range from the beginning of 770 nm excitation). The resulting time constant t was then used to calculate the rate (rate = 1/-t). The rates for each system are shown on **Table S1** and the resulting fits in **Figs. S9–S14**.

Table S1. *Rates of isomerization under 770 nm excitation (last entry under 660 nm excitation) with different solvents, sensitizers and sensitizer concentrations.*

**Solvent**	**Sensitizer**	**[Sensitizer], µM**	**Rate, min^{-1}**
**DCM**	I_2	50	0.16
**DCM**	I_2	100	0.39
**DCM**	I_2	200	1.1
**DCM**	I_2	300	1.8
**DCM**	I_2	400	2.7
**DCM**	I_2	500	3.5
**TCM**	I_2	200	0.52
**Hexane**	I_2	200	0.16
**DCM, degassed**	I_2	200	0.072
**DCM**	PdPc	0.36	0.0088
**DCM**	PdPc	50	0.096
**TCM**	PdPc	50	0.25
**DCM**	-	-	0.0032
**DCM**	I_2	5	0.0366
**DCM**, 660 nm exc	I_2	5	0.1597

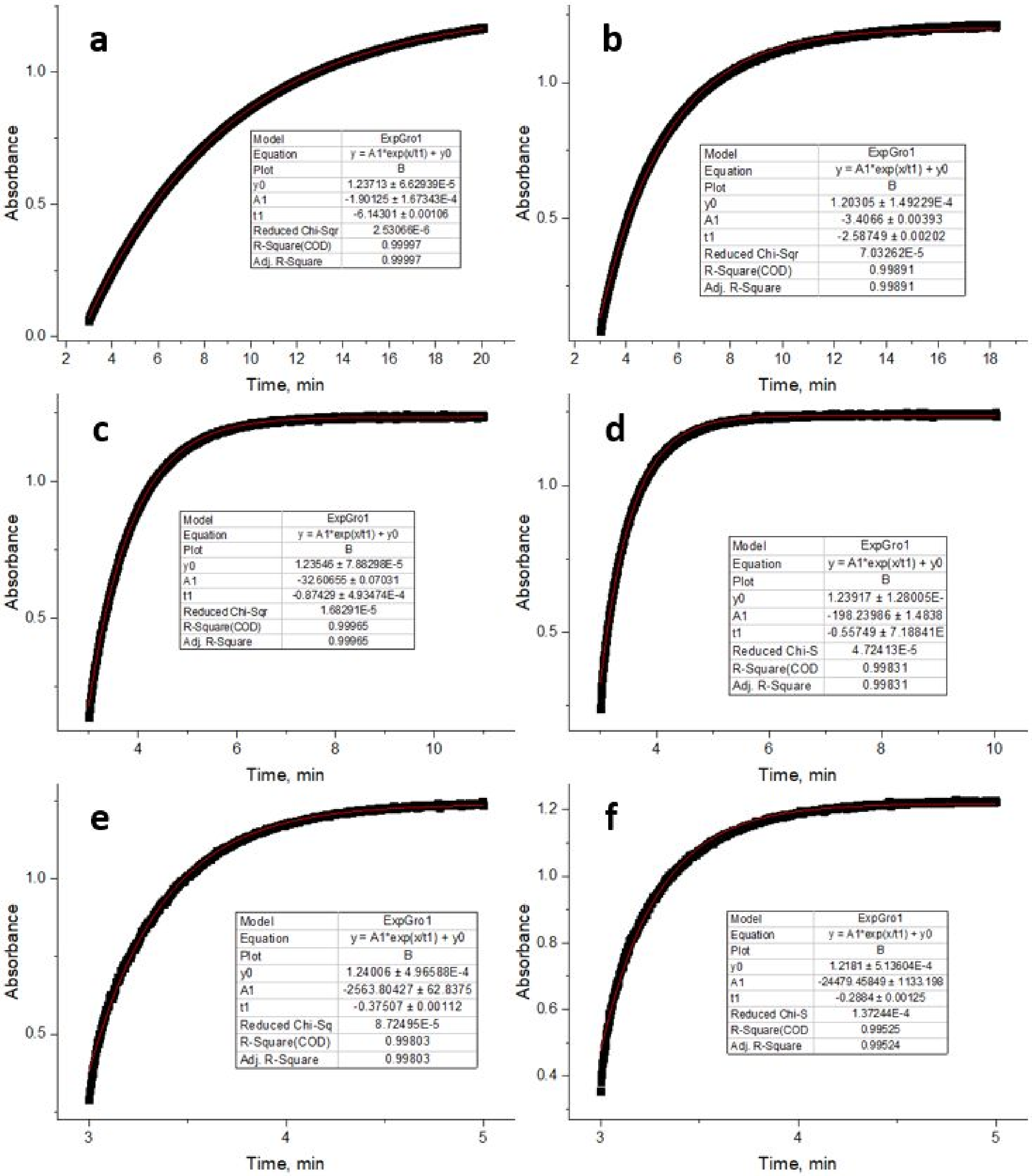

Figure S9. *Fittings of the photoisomerization curves of 50 µM of **1** in DCM with **a)** 50, **b)** 100, **c)** 200, **d)** 300, **e)** 400 and **f)** 500 µM of iodine.*

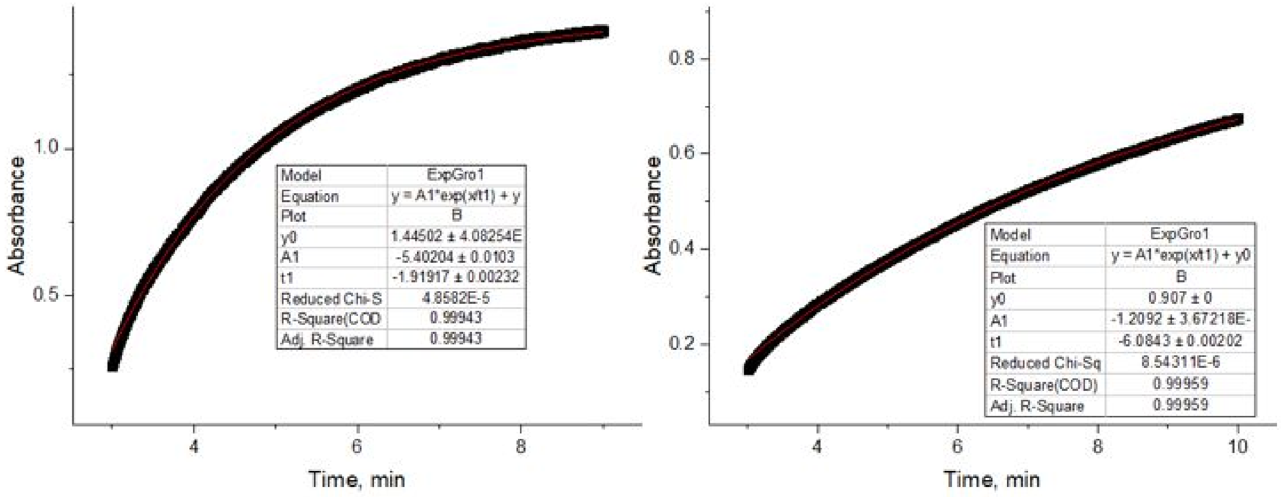

Figure S10. *Fittings of the photoisomerization curves of 50 µM of* ***1*** *in TCM (left) and hexane (right) with 200 µM of iodine.*

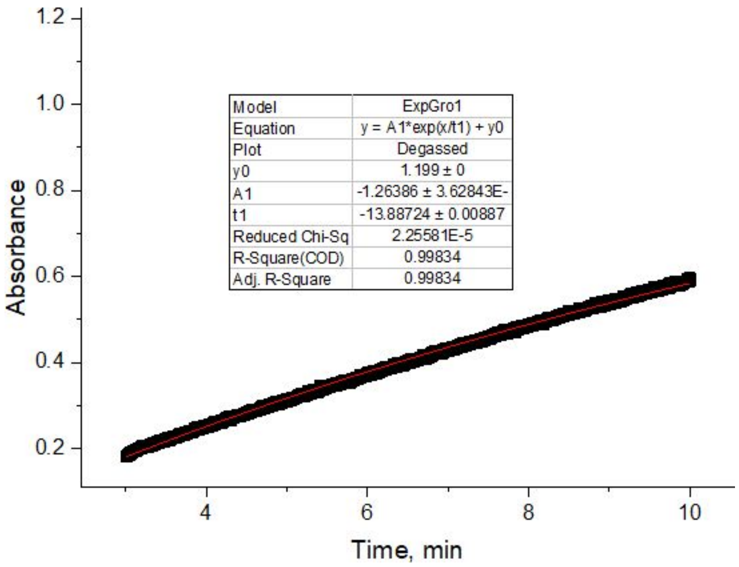

Figure S11. *Fitting of the photoisomerization curve of 50 µM of* ***1*** *in freeze-pump-thaw degassed DCM with 200 µM of iodine.*

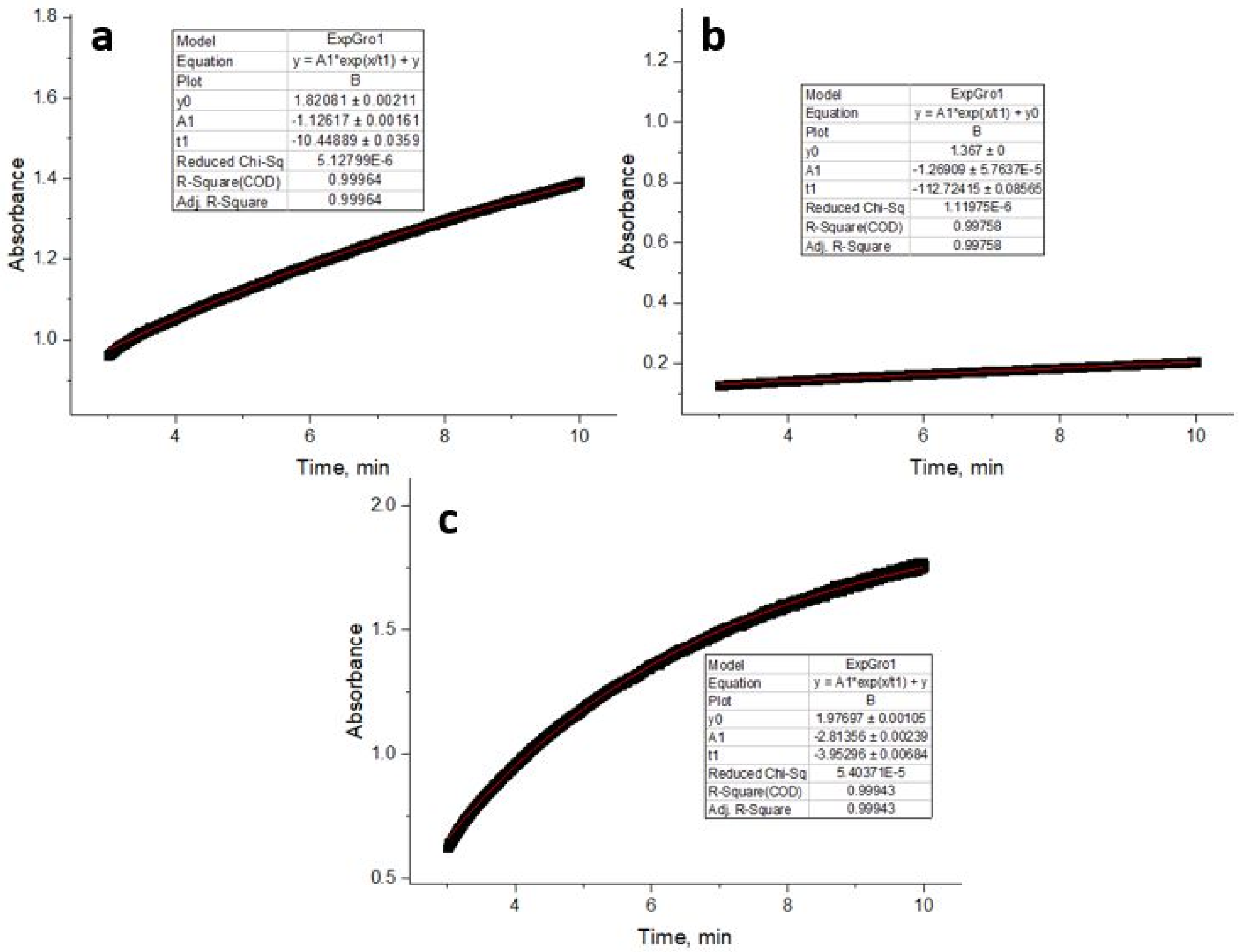

Figure S12. *Fittings of the photoisomerization curves of 50 µM of **1** in DCM with **a)** 0.36 µM and **b)** 50 µM of PdPc and **c)** in TCM with 50 µM of PdPc.*

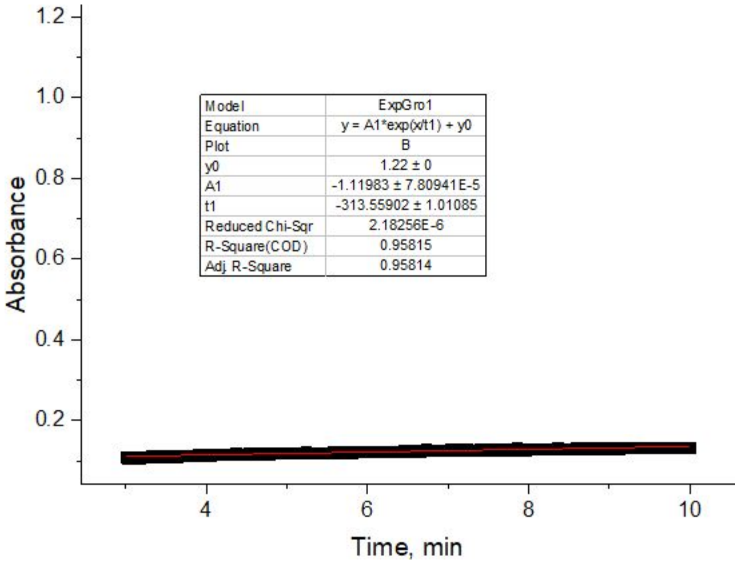

Figure S13. *Fitting of the thermal (dark) isomerization curve of 50 µM of **1** only in DCM.*

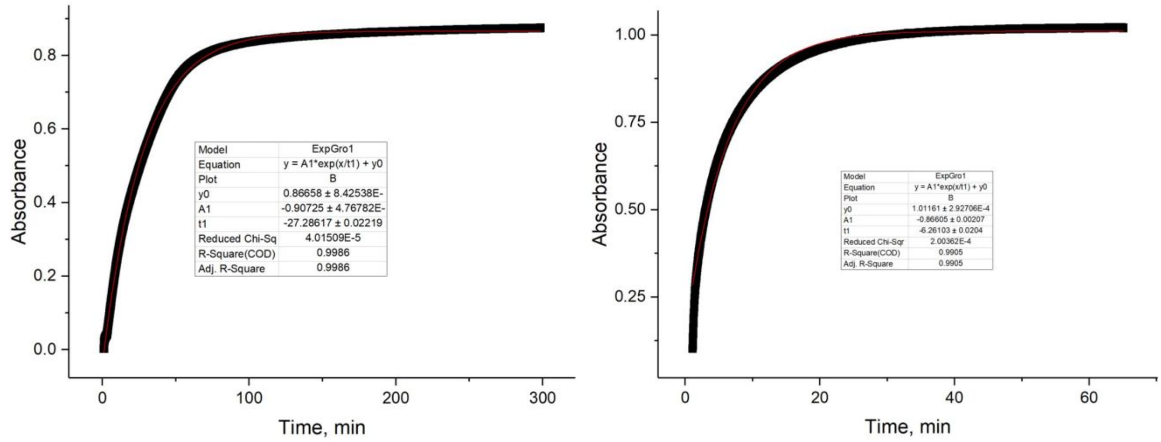

Figure S14. *Fittings of the photoisomerization curves of 50 µM of 1 in DCM with 5 µM of* I_2 *upon excitation with 770 nm (left) and 660 nm light (right).*

Materials

All solvents, reagents and other chemicals were purchased from Sigma-Aldrich, TCI Europe or VWR and used as provided unless stated otherwise. Reported synthetic procedures were slightly modified to prepare the well-known 4,4'-dimethoxyazobenzene[1,2] and PdPc[3]. Reactions were monitored with thin-layer chromatography (TLC) on commercial Merck Silica 60 F_{254} TLC plates, and the developed plates were visualized with UV irradiation (254 nm) or with potassium permanganate and cerium ammonium molybdate stains. Yields are isolated yields. Nuclear magnetic resonance spectra (NMR) were measured with a 500 MHz JEOL ECZR 500 instrument at 25 °C and processed with the JEOL Delta NMR software version 5.3.1 (Windows).

4,4'-dimethoxyazobenzene. 4-hydroxy-4'-methoxyazobenzene (400 mg, 1.74 mmol), sodium hydroxide (152 mg, 3.77 mmol) and iodomethane (1.08 ml, 17.5 mmol) were stirred in distilled water (7.3 ml) at 60°C overnight. The crude product was extracted with ethyl acetate and the organic phase was dried with magnesium sulfate and concentrated. Purification by column chromatography (15% ethyl acetate in n-hexane) yielded the product (342 mg, 81%) as an orange crystalline solid. ^{1}H NMR (500 MHz, $CDCl_3$) σ 7.87 (d, J = 9.2 Hz), 6.99 (d, J = 8.6 Hz), 3.88 (s, 6H). **(Fig. S15)**

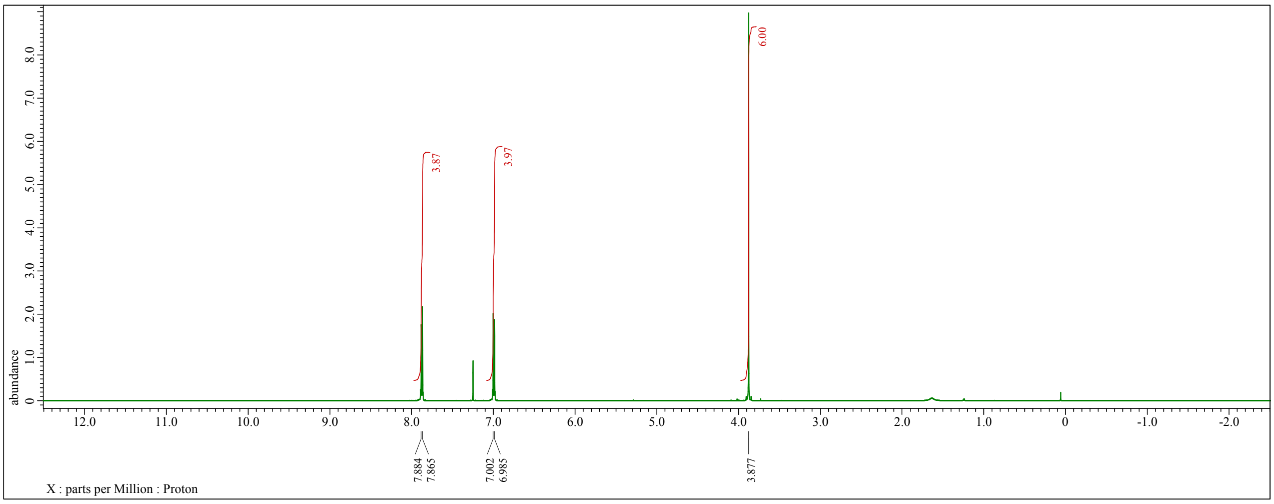

Figure S15. *^{1}H NMR spectrum of **1**.*

Diethyl 4,4'-azodibenzoate (4). Ethyl 4-aminobenzoate (1.0 g, 6.06 mmol), potassium permanganate (3.0 g, 19 mmol) and iron (II) sulfate (3.0 g, 10.8 mmol) were refluxed in dichloromethane for 48 hours. The reaction mixture was then filtered through silica and purified by column chromatography (dichloromethane) to yield **4** as an orange solid (317 mg, 32%). ^{1}H-NMR (500 MHz, $CDCl_3$) δ 8.19 (d, J = 8.6 Hz, 4H), 7.96 (d, J = 8.6 Hz, 4H), 4.40 (q, J = 7.1 Hz, 4H), 1.41 (t, J = 7.2 Hz, 6H). (**Fig. S16**)

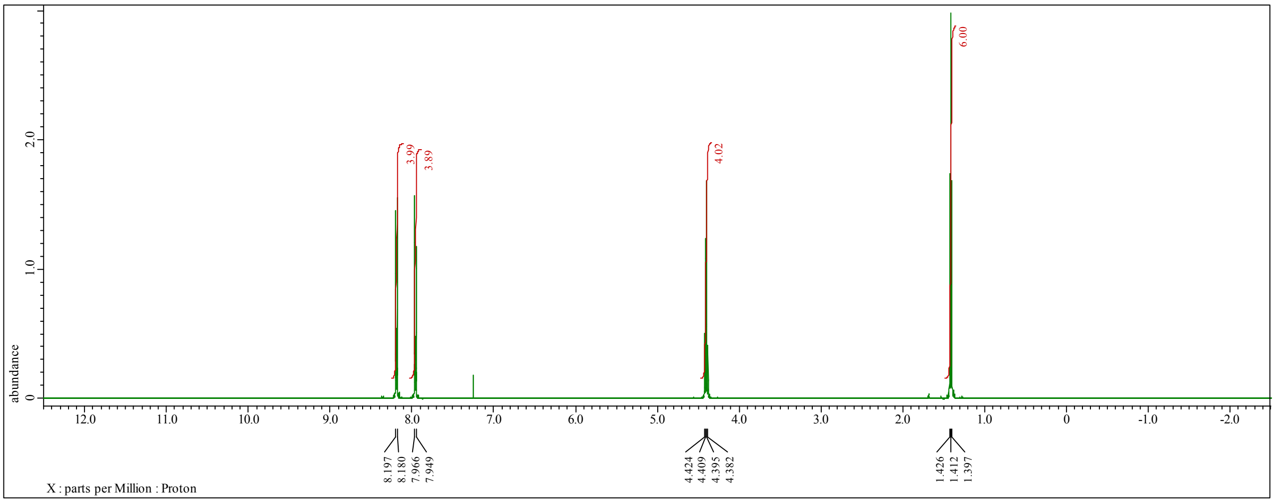

Figure S16. *^{1}H NMR spectrum of **4**.*

***N,N*-dimethyl-4-amino-4'-methoxyazobenzene (5)**. *N,N*-dimethyl-4-amino-4'-hydroxyazobenzene (100 mg, 0.415 mmol), methyl iodide (31 ul, 0.498 mmol) and potassium carbonate (115 mg, 0.83 mmol) were stirred in acetonitrile (1 ml) at room temperature for 19 hours. The reaction mixture was then diluted with ethyl acetate, washed with water, dried, concentrated and purified by column chromatography (dichloromethane) to yield **5** (61 mg, 57%) as an orange-yellow solid. ^{1}H-NMR (500 MHz, $CDCl_3$) δ 7.84 (d, J = 9.2 Hz, 4H), 6.99 (d, J = 8.6 Hz, 2H), 6.76 (d, J = 9.2 Hz, 2H), 3.87 (s, 3H), 3.07 (s, 6H). (**Fig. S17**)

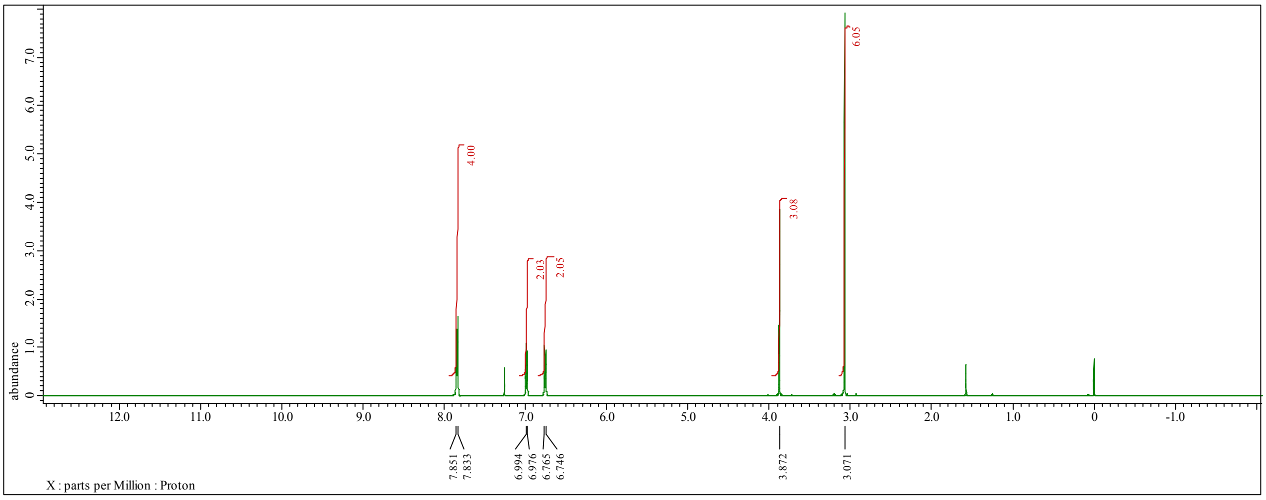

Figure S17. *^{1}H NMR spectrum of **5**.*

PdPc. 1,4,8,11,15,18,22,25-Octabutoxy-29*H*,31*H*-phthalocyanine (2*H*-Pc; 49 mg, 45.8 μmol) and palladium (II) acetate (11.3 mg, 50.4 μmol) were refluxed in *N,N*-dimethylformamide (8 ml) overnight. After confirming that a transformation had taken place with UV-Vis spectroscopy, the reaction mixture was diluted with chloroform, filtered, washed three times with distilled water, dried

with magnesium sulfate and concentrated. The crude product was further purified by column chromatography (0–20% ethyl acetate in dichloromethane) to yield the product (51.6 mg, 96%) as a green solid. ^{1}H NMR (500 MHz, $CDCl_3$) σ 7.55 (s, 8H), 4.86 (m, 16H), 2.24 (m, 16H), 1.64 (m, 16H), 1.08 (m, 24H). (**Fig. S18**) UV-Vis (10 μM): λ_{max} 734 nm (ε = 64,600 dm^3 mol^{-1} cm^{-1}). (**Fig. S19**)

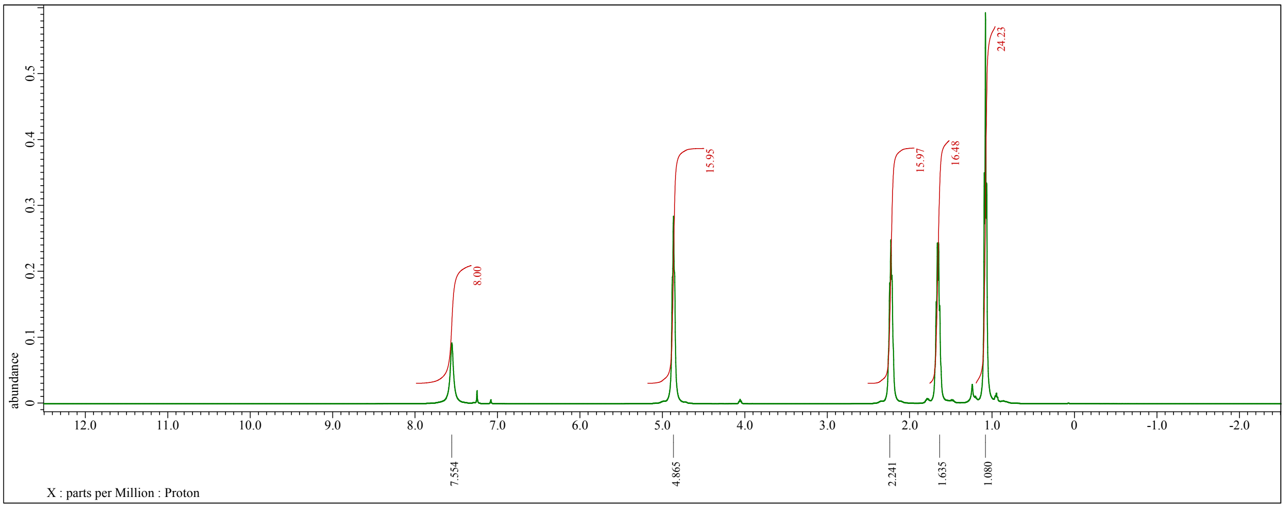

***Figure S18**. ^{1}H NMR spectrum of PdPc.*

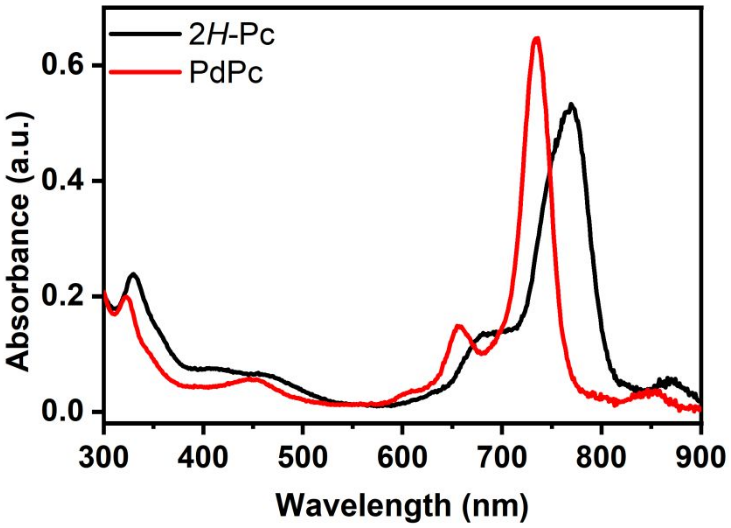

***Figure S19**. UV-Vis spectrum of PdPc (10 μM) and its precursor 2H-Pc (5 μM).*

References

(1) Bléger, D.; Schwarz, J.; Brouwer, A. M.; Hecht, S. O-Fluoroazobenzenes as Readily Synthesized Photoswitches Offering Nearly Quantitative Two-Way Isomerization with Visible Light. *J. Am. Chem. Soc.* **2012**, *134* (51), 20597–20600. https://doi.org/10.1021/ja310323y.

(2) Goulet-Hanssens, A.; Rietze, C.; Titov, E.; Abdullahu, L.; Grubert, L.; Saalfrank, P.; Hecht, S. Hole Catalysis as a General Mechanism for Efficient and Wavelength-Independent Z → E Azobenzene Isomerization. *Chem* **2018**, *4* (7), 1740–1755. https://doi.org/https://doi.org/10.1016/j.chempr.2018.06.002.

(3) Niemi, M.; Tkachenko, N. V; Efimov, A.; Lehtivuori, H.; Ohkubo, K.; Fukuzumi, S.; Lemmetyinen, H. Exciplex Mediated Photoinduced Electron Transfer Reactions of Phthalocyanine-Fullerene Dyads. *J. Phys. Chem. A* **2008**, *112* (30), 6884–6892. https://doi.org/10.1021/jp801498w.

(4) Dunphy, I.; Vinogradov, S. A.; Wilson, D. F. Oxyphor R2 and G2: Phosphors for Measuring Oxygen by Oxygen-Dependent Quenching of Phosphorescence. *Anal. Biochem.* **2002**, *310* (2), 191–198. https://doi.org/https://doi.org/10.1016/S0003-2697(02)00384-6.

(5) Vanderkooi, J. M.; Maniara, G.; Green, T. J.; Wilson, D. F. An Optical Method for Measurement of Dioxygen Concentration Based upon Quenching of Phosphorescence. *J. Biol. Chem.* **1987**, *262* (12), 5476–5482. https://doi.org/https://doi.org/10.1016/S0021-9258(18)45596-2.

(6) Sato, T.; Hamada, Y.; Sumikawa, M.; Araki, S.; Yamamoto, H. Solubility of Oxygen in Organic Solvents and Calculation of the Hansen Solubility Parameters of Oxygen. *Ind. Eng. Chem. Res.* **2014**, *53* (49), 19331–19337. https://doi.org/10.1021/ie502386t.
